# Increased Relative Risk of Tick-Borne Encephalitis in Warmer Weather

**DOI:** 10.3389/fcimb.2018.00090

**Published:** 2018-03-22

**Authors:** Milan Daniel, Vlasta Danielová, Alena Fialová, Marek Malý, Bohumír Kříž, Patricia A. Nuttall

**Affiliations:** ^1^Centre for Epidemiology and Microbiology, National Institute of Public Health, Prague, Czechia; ^2^Department of Biostatistics, National Institute of Public Health, Prague, Czechia; ^3^Third Medical Faculty, Charles University, Prague, Czechia; ^4^Department of Zoology, University of Oxford, Oxford, United Kingdom; ^5^Centre for Ecology and Hydrology, Wallingford, United Kingdom

**Keywords:** tick-borne encephalitis, TBEV, *Ixodes ricinus*, arbovirus, seasonality, climate change

## Abstract

Tick-borne encephalitis (TBE) is a serious acute neuroinfection of humans caused by a tick-borne flavivirus. The disease is typically seasonal, linked to the host-seeking activity of *Ixodes ricinus* (predominantly nymphs), the principal European tick vector species. To address the need for accurate risk predictions of contracting TBE, data on 4,044 TBE cases reported in the Czech Republic during 2001–2006 were compared with questing activity of *I. ricinus* nymphs monitored weekly at a defined location for the same 6-year period. A time shift of 21 days between infected tick bite and recorded disease onset provided the optimal model for comparing the number of cases of TBE with numbers of questing nymphs. Mean annual distribution of TBE cases and tick counts showed a similar bimodal distribution. Significantly, the ratio of TBE cases to questing nymphs was highest in the summer-autumn period even though the number of questing nymphs peaked in the spring-summer period. However, this pattern changed during a period of extreme meteorological events of flooding and abnormally high temperatures, indicating that changes in climate affect the incidence of TBE. Previous studies failed to link human behavior with changes in incidence of TBE but showed extrinsic temperature impacts arbovirus replication. Hence, we hypothesize the apparent discrepancy between peak nymphal tick activity and greatest risk of contracting TBE is due to the effect of temperature on virus replication in the tick vector. Relative proportions of questing nymphs and the numbers of weeks in which they were found were greater in summer-autumn compared with spring-summer at near-ground temperatures >5°C and at standard day and weekly average temperatures of >15°C. Thus, during the summer-autumn period, the virus dose in infected tick bites is likely greater owing to increased virus replication at higher microclimatic temperatures, consequently increasing the relative risk of contracting TBE per summer-autumn tick bite. The data support the use of weather-based forecasts of tick attack risk (based on daytime ambient temperature) supplemented with weekly average temperature (as a proxy for virus replication) to provide much-needed real-time forecasts of TBE risk.

## Introduction

Tick-borne encephalitis (TBE) is a serious acute neuroinfection of humans caused by a member of the virus family, Flaviviridae. The principal vector of the European subtype of TBE virus (TBEV) is the most epidemiologically important European tick species, *Ixodes ricinus*. In 1948, across several areas of the Czech Republic, 56 cases of a viral neuroinfection were reported linked to tick-bite (Ružek, 2015); TBEV was isolated from the patients and from ticks collected in Central Bohemia and Moravia (Gallia et al., 1949; Krejčí, 1949; Rampas and Gallia, 1949). In subsequent years, TBE was recognized in other European countries and the virus isolated: Hungary (Fornosi and Molnár, 1952), Poland (Przesmycki et al., 1954), Bulgaria (Vaptsarov et al., 1954), Slovenia (Bedjanič et al., 1955), Austria (Pattyn and Wyler, 1955), Romania (Draganescu, 1959), Finland (Oker-Blom, 1956), Germany (Sinnecker, 1960), and Sweden (Kaäriainen et al., 1961). Presently in Europe, TBE is most prevalent in Southern Germany, Switzerland, Austria, the Czech Republic, Slovakia, Hungary, Slovenia, the Baltic countries, Poland, parts of Scandinavia, and Russia (Heinz et al., 2013). Longitudinal surveillance in Austria shows TBE emergence in previously unaffected regions consistent with similar findings in Norway, Sweden, and Denmark (Skarpaas et al., 2004; Johan et al., 2006; Fomsgaard et al., 2009; Heinz et al., 2013). During the period of our study (2001–2006), TBE mortality rate in the Czech Republic was 0.2% (EPIDAT, see below). Patients required hospitalization for 3–4 weeks followed by 6–8 weeks or more rehabilitation (Duniewiecz, 1999).

Since the first virus isolation, particular attention is paid to TBEV and its vector in the Czech Republic. As early as 1954, in the first European monograph on TBE, seasonal bimodal curves of weekly registered TBE cases and tick occurrence were recorded and the influence of meteorological factors was recognized (Raška and Bárdoš, 1954). Owing to the relatively high number of cases, TBE became a notifiable disease in the Czech Republic in 1970. Since 1971, records of laboratory confirmed cases are collated in an extensive database (EPIDAT, National Institute of Public Health, Prague), which includes clinical data on TBE cases as well as probable date and place of infection, and time of onset of symptoms. This unique database, comprising 21,847 TBE cases reported in 1970–2016, enables the evaluation of temporal changes in the epidemiology of TBE over several decades coinciding with major changes in climatic variables, and including regional analysis (Daniel et al., 2011; Kříž et al., 2012). Since the 1990s, increasing incidences of TBE have been reported in several Central and Western European countries. In 2012, TBE became an obligatory notifiable disease in all countries of the European Union (2012/506/EU: Commission Implementing Decision of 8 August 2012), with a unified case definition (Amato-Gauci and Zeller, 2012). As a consequence of the increasing incidence, a detailed study of the seasonal dynamics of *I. ricinus* tick activity was carried out in 2001–2006 within the framework of the WHO/EC project, Climate Change and Adaptation Strategies for Human Health (Menne and Ebi, 2006). Host-seeking activity of nymphs (epidemiologically the most important stage), and concurrent near-ground temperature and relative air humidity, were recorded 1 day each consecutive week for 6 years, and the data compared with standard weather data collected at a nearby meteorological station (Daniel et al., 2015). Temperature was shown to be the strongest predictor of the seasonality of tick host-seeking activity. These results are the basis of a computer model for predicting the risk of tick attack in the Czech Republic. Predictions are actualized according to routine weather forecasts modified by the 3–4 day synoptic situation, and publicized by the Czech Hydrometeorological Institute (CHMI) with risk categorized at 10 different levels (Daniel et al., 2010).

Given that only low numbers of ticks are infected with TBEV (see section Discussion), comparatively few people bitten by ticks develop clinical neurological disease (Duniewiecz, 1999). This also applies to people bitten by infected ticks. For example, in a highly active natural focus of TBEV transmission, the ratio of symptomatic to inapparent TBE cases was approximately 2:3, representing 6.1 and 9.6%, respectively of permanent residents (Luňáčková et al., 2003). Hence current predictions are of tick attack risk rather than of the risk of TBE clinical infections. Here we examine the relationship between abundance of questing nymphal ticks and incidence of TBE in order to test if weather-based forecasts of tick attack risk can be used to predict risk of contracting TBE.

## Materials and methods

### Incidence of TBEV clinical infections in humans

Since 1970, laboratory-confirmed cases of TBE in the Czech Republic have been recorded in the EPIDAT database (http://www.szu.cz/publikace/data/infekce-v-cr), a national reporting system maintained by the National Public Health Institute (NIPH) in Prague, Czech Republic. Each case of TBE is reported by the diagnosing physician to the public health authorities, which then undertake epidemiological investigations. Through an interview with the patient, records are made of medical history, probable time and place of infection, and possible transmission route. These data are then collated on a weekly basis by NIPH. For our study, 4,044 cases of TBE registered in the Czech Republic during 2001–2006 were classified according to the date of the first recorded clinical symptoms.

### Monitoring *Ixodes ricinus* tick activity

The tick collecting site and monitoring design to determine the seasonal variation in *I. ricinus* questing activity have been described previously (Daniel et al., 2015). Briefly, *I. ricinus* host-questing activities were investigated in 2001–2006 by the standard flagging method in a defined site in the southern outskirts of Prague, 4 km from the main observatory station of the CHMI. Ticks were counted at weekly intervals from March to November. Altogether 208 monitoring visits were conducted. Ticks were not removed from the plots but were immediately released back to the place of collection. Detailed numbers of ticks counted and concurrent records of microclimate temperature are published (Daniel et al., 2015). The 6-year study period allowed time for the development of three tick generations (the life cycle of *I. ricinus* typically takes 2 years to complete in Central Europe) (Daniel and Dusbábek, 1994). Only data for nymphs were used as this stage poses the greatest risk to humans (Lindblom et al., 2013).

### Meteorological data

Three sets of temperature data were used for comparisons of meteorological conditions with *I. ricinus* host-questing activity:

1. Near-ground temperature measured using a mercury thermometer placed at 1 cm above the ground, in shadow and away from the sun (Daniel et al., 2015).
2. Standard day temperature recorded by the nearby CHMI observatory on the same day and at the same time as the near-ground temperature was recorded in the tick monitoring site.
3. Weekly average temperature for each week of the 2001–2006 monitoring period obtained from the CHMI database.

Near-ground temperature represents the real-time ecoclimatic temperature that ticks experience in the monitored site. Such measurements are not routinely available and therefore temperature measurements recorded daily by standard meteorological methods are used for tick questing activity forecasts. However, weekly average temperature better characterizes the temperature influence on virus replication in infected ticks (Danielová, 1975).

### Statistical analysis

To compare meteorological conditions and tick counts with the estimated times of human infection (i.e., infected tick bite), the predictor variables were lagged by performing a time shift (1–6 weeks) to take into account the incubation period between virus infection and clinical symptoms. The best model was selected using two different approaches, Akaike's and Bayesian information criteria, which measure relative quality of statistical models for given data. They take into account both the overall fit of the model (statistical goodness of fit measured via log-likelihood) and the number of parameters that have to be estimated to achieve the particular degree of fit, by imposing a penalty for increasing the number of parameters.

For statistical testing, square root transformation was applied to the data for tick activity and TBE cases to control for heteroskedasticity and obtain a near-normal distribution. Statistical analysis was based on the linear regression model to test the linear relationship between square root transformed counts of ticks/TBE cases. The model for each respective year is expressed as:

(1) $$\begin{array}{r} y_{i}\;\sim \;\beta_{0}+\beta_{1}x_{i}+\beta_{2}P_{i}+\beta_{3}{x_{i}P}_{i},\;i=1,2,\ldots ,n \end{array}$$

where

*y*_*i*_ is the square root of the number of TBE cases

*x*_*i*_ is the square root of the number of active nymphs

$P_{i}=\{\;\begin{array}{c} 0\text{ if an observation belongs to spring}-\text{summer period }\\ 1\text{ if an observation belongs to summer}-\text{autumn period} \end{array}\;$

β_0_, β_1_, β_2_, β_3_ are regression coefficients

*n* is the number of analyzed weeks in the year.

Difference of the slopes for spring-summer (β_1_) and summer-autumn (β_1_ + β_3_) period was tested via the regression coefficient β_3_ for the interaction term between the period indicator *P*_*i*_ and nymphal tick counts. Chow's test was used to examine whether both parameters of one regression line are equal to those of another regression line. Temperature and the respective numbers of ticks were grouped into categories and the cumulative frequencies were calculated. The statistical significance level was set to 5%. Data were processed using R software, version 3.1.2 (R Core Team, 2014).

All data relevant to the conclusions of this manuscript are available upon request to the authors.

## Results

### Seasonal incidence of TBE

**Figure 1:**
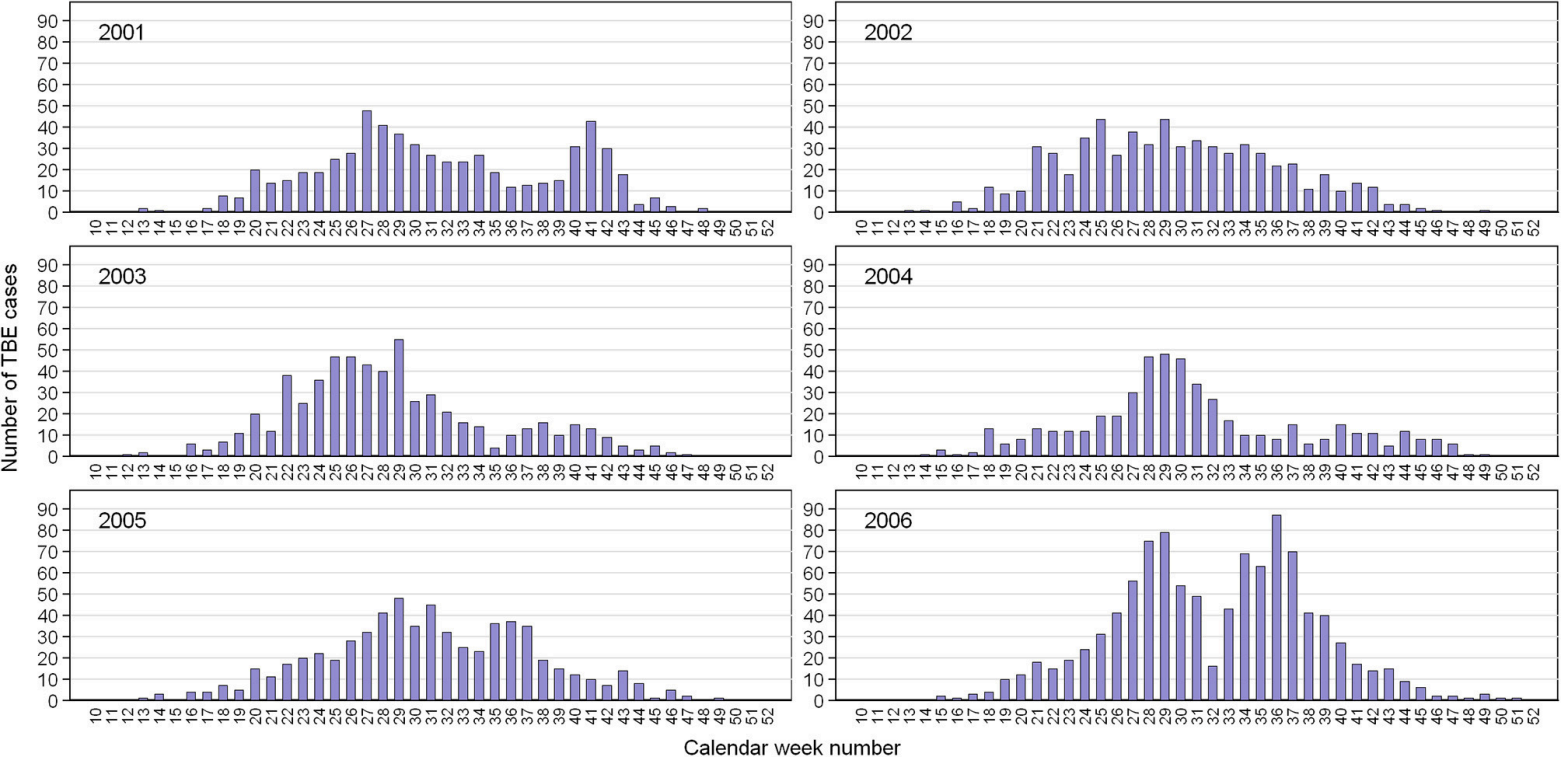
Cases of TBE in the Czech Republic by calendar week of onset of clinical symptoms. Data from the Czech EPIDAT database for 2001–2006.

During 2001–2006, 4,044 cases of TBE were reported in the Czech Republic. Of these, 21 cases (0.52%) were attributed to alimentary infections from drinking/eating contaminated milk products (Kříž et al., 2009). The rest were assumed to have resulted from the bite of an infected *I. ricinus* tick. Determination of the seasonal incidence of infection was based on the distribution of cases by calendar week of the onset of symptoms with an accuracy of 1 week (Figure 1). Generally, the seasonal incidence of cases is characterized by a bimodal curve, as seen across much of Europe (European Centre for Disease Prevention and Control, 2016). In 2001, the late summer peak was almost as high as the spring peak while in 2006, the late summer peak was higher than the spring peak.

### Comparison of the seasonal incidence of TBE and the abundance of host-questing *I. ricinus* nymphs

**Figure 2:**
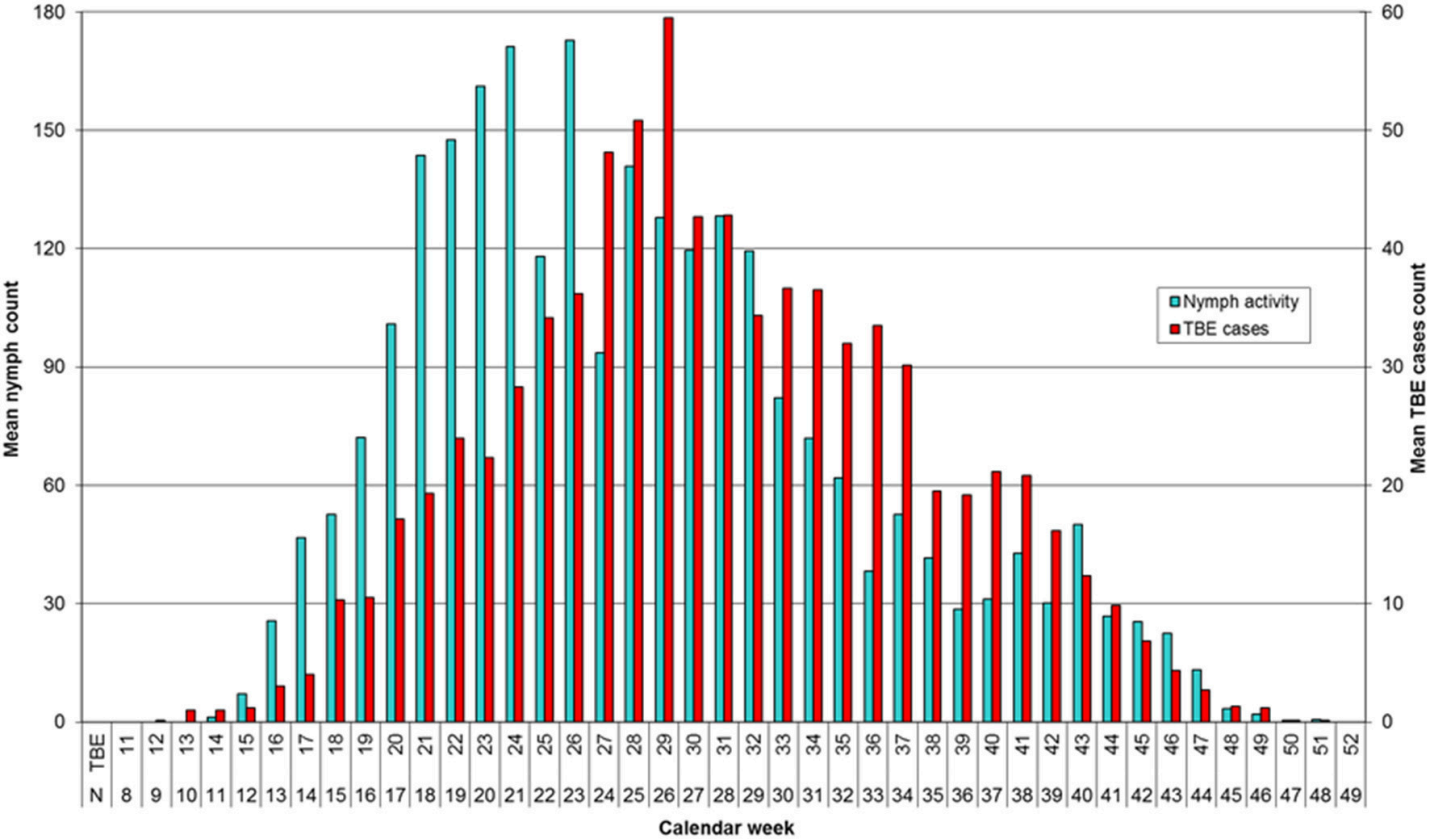
Seasonal activity of *Ixodes ricinus* nymphs by week of collection and incidence of TBE by week of onset. Data for 2001–2006 are compared using a 3-week shift in alignment. Spring-summer and summer-autumn periods are distinguished by the sharp drop in number of questing nymphs around week 22 and fall in near ground temperature to 10–12°C (Daniel et al., 2015).

Mean annual distribution of TBE cases and tick counts show a similar bimodal profile (Figure 2). However, whereas the number of questing nymphs reached a maximum in the spring-summer period (~week 21) and then declined but with a minor second peak in autumn (~week 40), the peak incidence of TBE was at the beginning of the summer-autumn period (~week 29), and showed a slower rate of decline compared to nymph activity at the end of the summer period and a relatively higher summer-autumn peak (~week 40).

**Figure 3:**
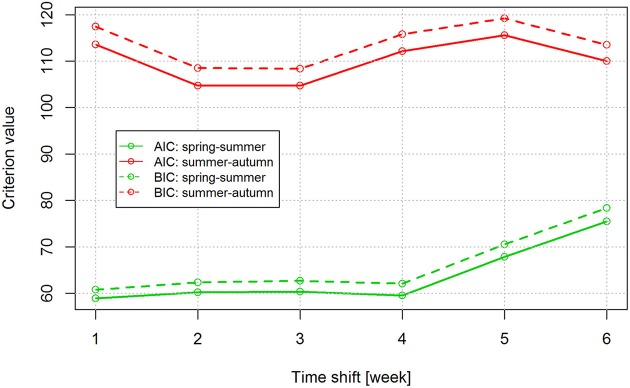
Analysis of the effect of a 1–6 week time shift on the estimated time of infection using Akaike's (AIC) and Bayesian (BIC) information criteria. The two criteria were used to select the best model in the group of models (1–6 week shift) compared. Lower criterion values indicate the preferred model.

To investigate the apparent seasonal discrepancies, the linear regression relationship between the mean annual numbers of TBE cases and tick abundance was compared between the spring-summer and summer-autumn periods. Time shifts of 1–6 weeks were tested to account for the delay between time of infection and onset of TBE symptoms (Figure 3). There was a high degree of correlation in weekly count of TBE cases and weekly count of questing nymphs; the highest mean correlation was obtained with a 3-week shift (*r* = 0.95). However, even allowing for a 3-week delay between the time of an infected tick bite and the onset of clinically apparent disease, discrepancies were apparent in the seasonal dynamics of nymphal tick abundance and TBE incidence (Figure 2).

**Figure 4:**
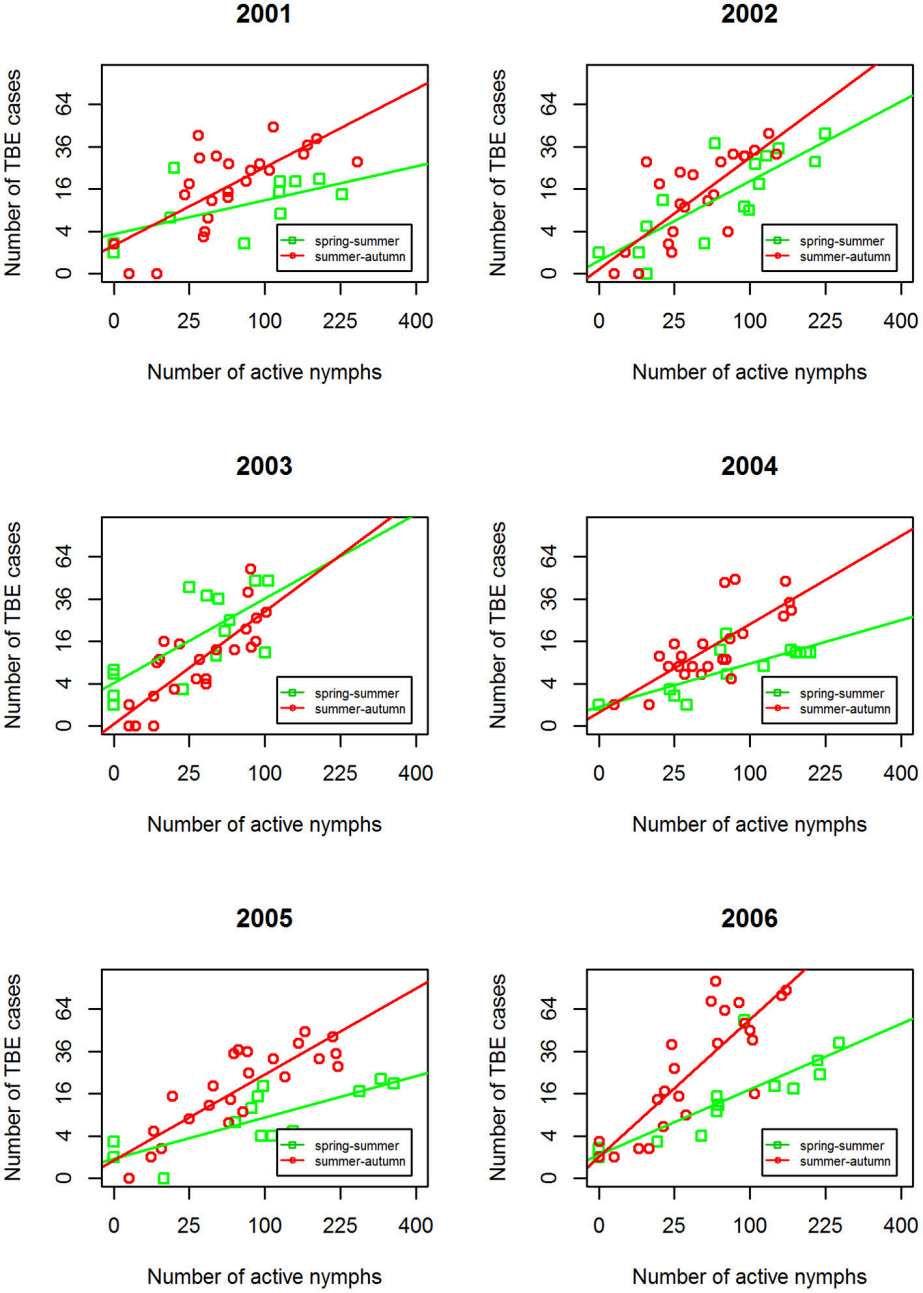
Inter-seasonal comparison of the linear regression relationship between numbers of host-questing *Ixodes ricinus* nymphs and cases of TBE. Square root transformation was used for both axes together with a 3-week shift in alignment.

Comparison of annual regression lines and their slopes showed that summer-autumn was consistently steeper than spring-summer (Figure 4, Supplementary Table 1). Over the 6-year time span, the difference in the slope of the regression lines between the two seasonal periods was highly significant (*p* < 0.001) indicating that the incidence rate of TBE grew more rapidly in the summer-autumn period than in the spring-summer period in response to the unit increase in tick questing rate. The greatest difference was observed for 2006 (*p* = 0.005) (Supplementary Table 1), reflecting the remarkably high relative incidence of TBE in the summer-autumn period (Figure 1). Even when 2006 was removed from the model, the difference between regression slopes for spring-summer and summer-autumn remained significant (*p* = 0.012). Seasonal regression lines within 2002 and 2003 were similar (*p* = 0.19 and *p* = 0.31, respectively); however, in 2003 (the year following extensive flooding), they were reversed indicating there were relatively fewer cases of TBE per given number of questing ticks during the summer-autumn period compared with the spring-summer period. This anomaly was probably a consequence of floods and unusual meteorological conditions (see section Discussion).

**Figure 5:**
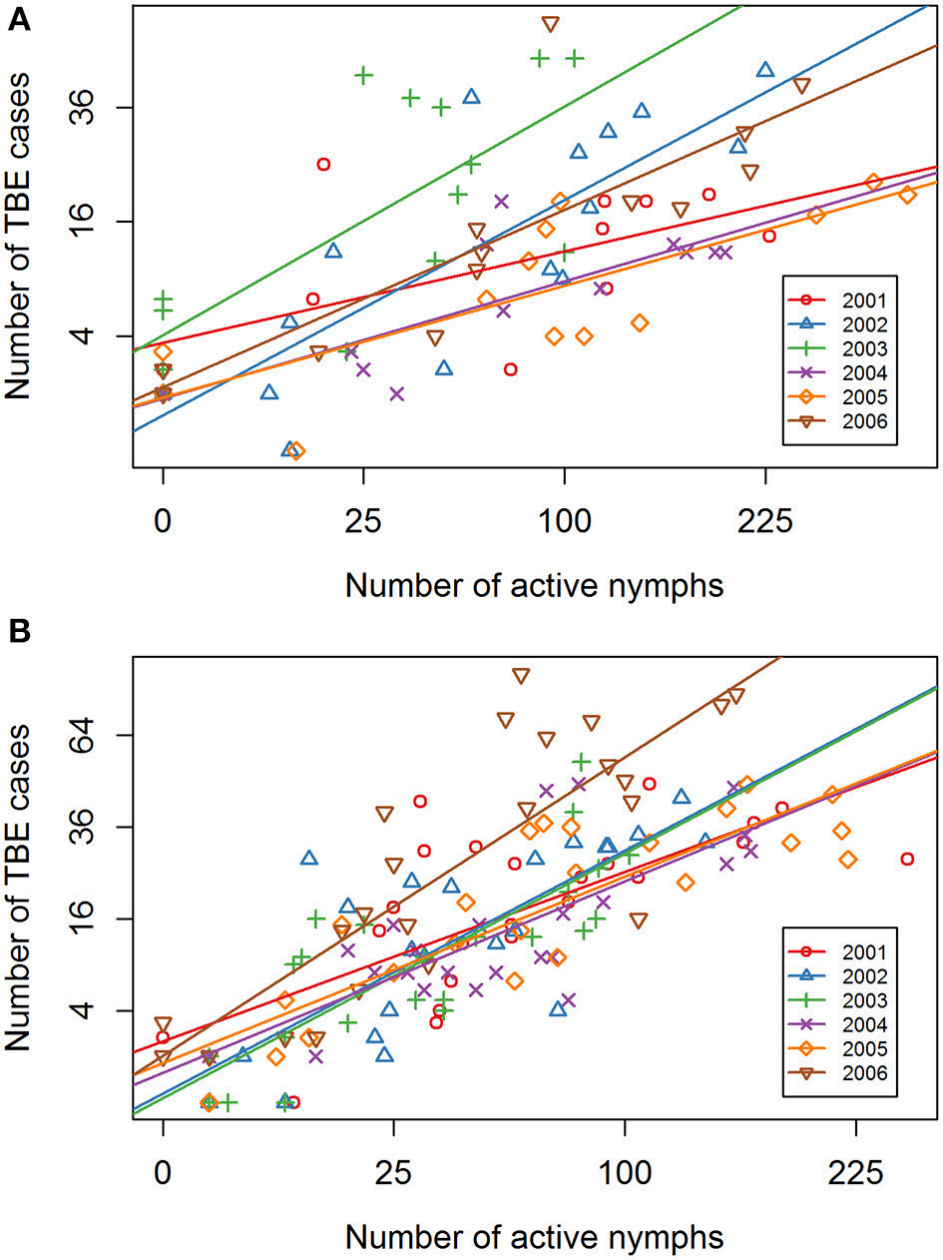
Inter-annual comparison of the linear regression relationship between numbers of host-questing *Ixodes ricinus* nymphs and cases of TBE. Year-on-year comparison for 2001–2006 of the regression lines for **(A)** the spring-summer period, and **(B)** the summer- autumn period. Square root transformation was used for both axes with a 3-week shift in alignment.

Year-on-year variability in the relationship between *I. ricinus* abundance and TBE incidence was greater in spring-summer period compared with summer-autumn (Table 1). This inter-seasonal difference reflects greater variability in meteorological conditions during spring. All regression lines in the summer-autumn period were similar except 2006 (Figure 5).

**Table 1:** Differences between numbers of ticks and TBE cases.

**Year**	**2001**	**2002**	**2003**	**2004**	**2005**	**2006**
**SPRING-SUMMER PERIOD**						
2001	–					
2002	0.075^*^	–				
2003	0.010	0.022	–			
2004	0.339	0.012	<0.001	–		
2005	0.332	0.005	<0.001	0.976	–	
2006	0.196	0.777	0.038	0.022	0.013	–
**SUMMER-AUTUMN PERIOD**						
2001	–					
2002	0.407	–				
2003	0.303	0.978	–			
2004	0.626	0.462	0.506	–		
2005	0.806	0.428	0.403	0.920	–	
2006	<0.001	0.002	0.001	<0.001	<0.001	–

* *p-values from Chow's test of whether the coefficients are equal in pairwise regression line comparisons of abundance of host-questing nymphs and cases of TBE*.

### Host-questing activity of *I. ricinus* nymphs and temperature: comparison of spring-summer and summer-autumn periods

**Figure 6:**
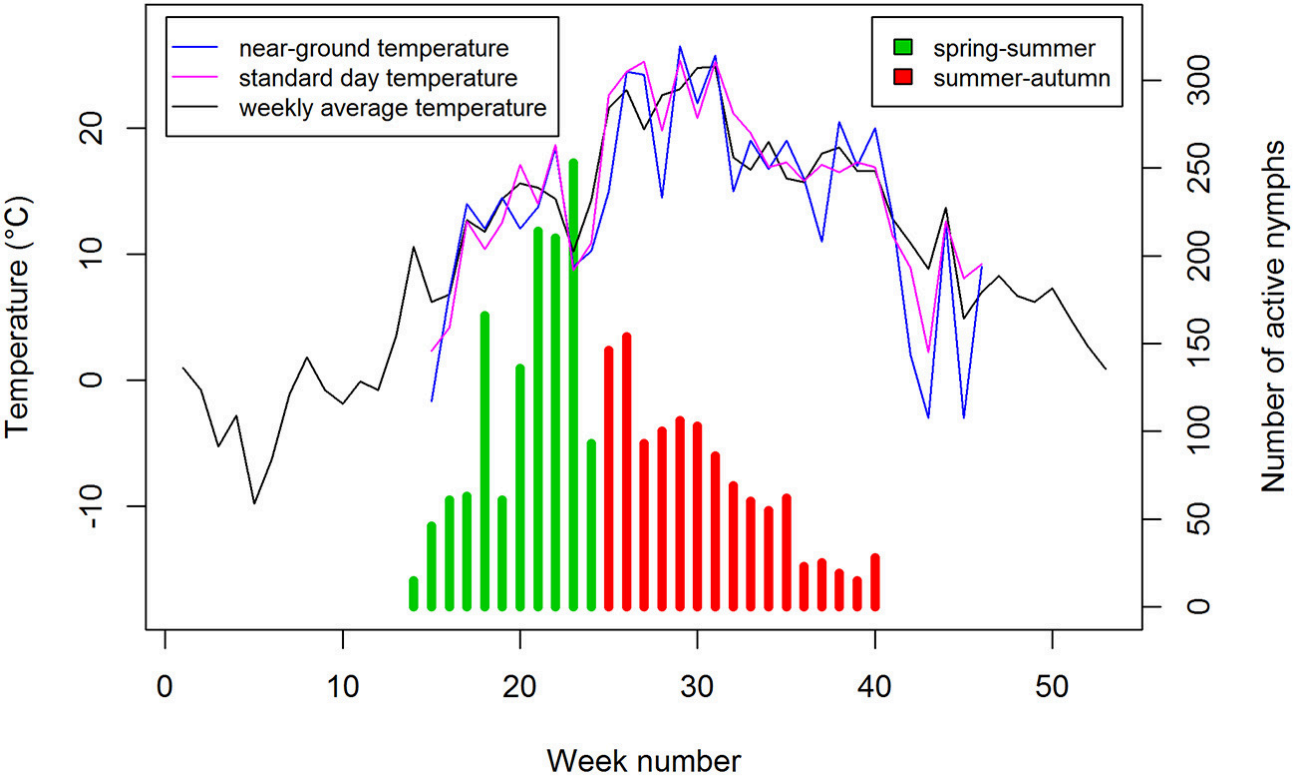
Host-questing *Ixodes ricinus* nymphs in spring-summer and summer-autumn compared with near-ground, standard day, and weekly average temperatures. Data for 2006.

Our previous study showed air temperature to be the best predictor of nymph questing activity compared with relative humidity or day length (Daniel et al., 2015), as was found in a study of the interaction between TBE incidence, ambient temperature and precipitation rate (Kříž et al., 2015)_._ Hence the variation in relationship between the numbers of questing *I. ricinus* nymphs and the number of TBE cases for the spring-summer and summer-autumn periods was examined in relation to ambient air temperature to which host-seeking ticks were exposed. Questing activity was examined in ranges of 5°C, comparing near-ground temperature in the monitoring site with standard day and weekly average temperatures recorded at the nearby CHMI observatory. Standard day and weekly average temperatures were generally 4–5°C higher than near-ground temperatures with a maximum difference of 10°C (in 2006; Figure 6). During the total 66 weeks of the spring-summer period for the 6-year period examined, 6,903 questing nymphs were recorded, whereas 7,955 questing nymphs were counted during the total 102 weeks of the summer-autumn period (Supplementary Table 2).

**Figure 7:**
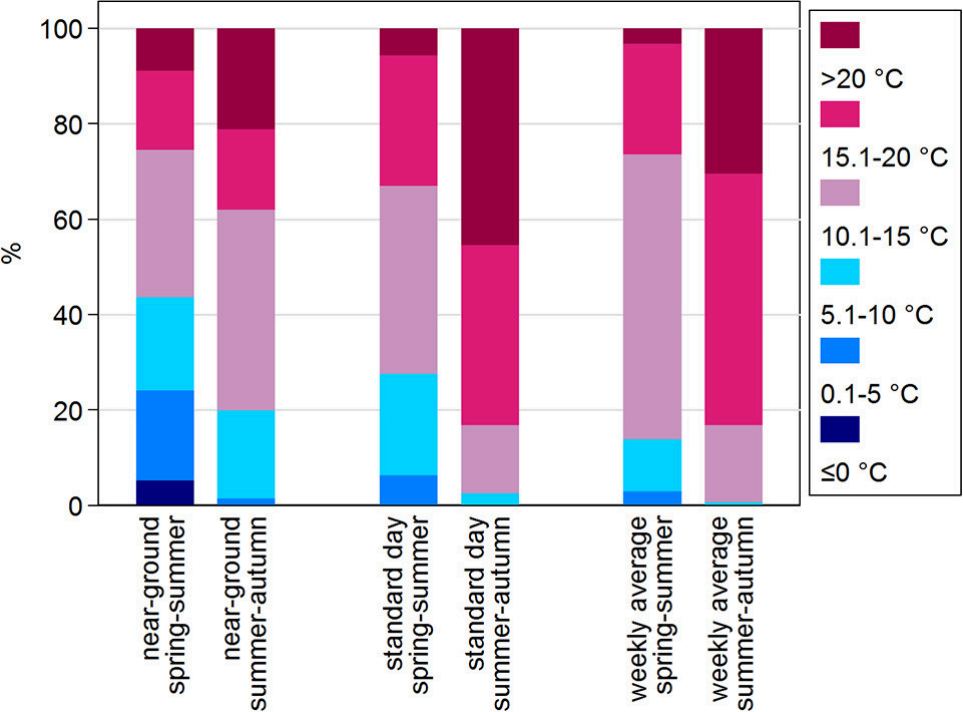
Proportions of host-questing *Ixodes ricinus* nymphs active at different temperature categories in spring-summer compared with summer-autumn periods. Proportions are expressed as % questing nymphs evaluated against near-ground, standard day, and weekly average temperatures.

**Figure 8:**
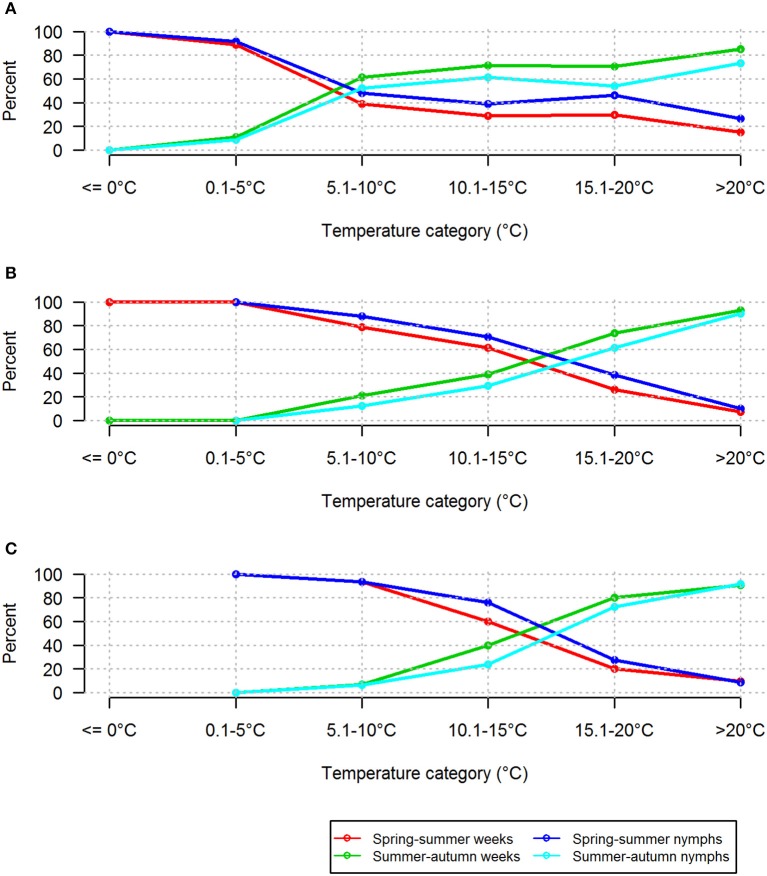
Relative proportions of nymphs and numbers of weeks in which they were found at different temperature categories in spring-summer and summer-autumn. Based on **(A)** near-ground temperature measured in the tick habitat, **(B)** standard day temperature measured by the CHMI observatory, and **(C)** weekly average of temperature derived from the CHMI database.

Based on near-ground temperatures, the proportions of questing nymphs recorded in the two lowest temperature ranges (≤0°C and 0.1–5°C) differed substantially between the two seasonal periods (Figure 7). These low temperature ranges were comparatively rare in the summer-autumn period (Supplementary Table 2). In the categories 5.1–10 and 10.1–15°C, similar proportions of nymphs were questing in the two seasonal periods although there were more records for the summer-autumn period. However, there was a marked difference in the proportions and numbers of nymphs questing in the higher temperature ranges (15.1–20°C and >20°C) during summer-autumn compared with spring summer although, not surprisingly, there were more records in these categories (Supplementary Table 2). This difference was more striking when comparisons were made using standard day and weekly average temperatures (Figure 7). The relative proportions of questing nymphs and the numbers of weeks in which they were found were greater in summer-autumn compared with spring summer at near-ground temperatures >5°C, and at standard day and weekly average temperatures of >15°C (Figure 8).

## Discussion

Although most cases of TBE arise from an infected tick bite, the ratio of symptomatic human TBE infections to questing *I. ricinus* nymphs was found to differ significantly between spring-summer and summer-autumn periods. The seasonal difference is apparent even when allowance is made for the interval between infected tick bite and the first symptoms of TBE. This interval includes the incubation period for TBE, which is highly variable between individuals partly because of the typical bi-phasic clinical course. The first phase, characterized by non-specific influenza-like symptoms with an incubation period of 3–21 days, may be missed in up to one third of cases and disease recognized directly with neurological symptoms (Duniewiecz, 1999). Other factors influencing the incubation phase include age, overall state of physical and mental health, and the virus dose transmitted by tick bite (Duniewiecz, 1999; Penyevskaya, 2008). Modeling indicated that 21 days was the best estimate of the time between infected tick bite and onset of TBE.

Given the need for accurate risk predictions of contracting TBE, we investigated the apparent discrepancy between seasonal incidence of TBE and abundance of host-questing nymphs. Our previous study showed near-ground temperature in the site of tick monitoring is the strongest predictor of tick activity, and showed a high correlation with local meteorological observations in Central Bohemia (Daniel et al., 2015). Similar observations apply to monitoring sites in northern Bohemia, northern Moravia, southern Bohemia, and in the Bohemian-Moravian Highlands (Daniel et al., 2016; Brabec et al., 2017). Hence seasonal discrepancies between numbers of active *I. ricinus* nymphs and TBE cases appear to be independent of locality. Likewise, the seasonal discrepancy in tick activity and TBE incidence is not explained by human activity (which has changed in the last 20 years), as exemplified by the rise in TBE cases at higher altitudes in the Czech Republic where land use and socio-economic conditions have remained unchanged (Daniel et al., 2011). Although promotion of healthy lifestyles encourages outdoor activity, greater environmental management reduces tick contact; furthermore, sustained national awareness campaigns have increased awareness of tick-borne diseases and preventative measures (Daniel et al., 2006, 2010). TBE vaccination in Austria of 85% of the population corresponds with a reduction in the number of TBE cases to ~16% of pre-vaccination levels; however, in the Czech Republic TBE vaccination only reached 23% coverage in 2013 (Heinz et al., 2013; Prymula, 2015). Although it cannot be ruled out that human activity accounts for the greater relative risk of contracting TBE during summer-autumn, the lack of any clear evidence suggests an alternative explanation.

Seasonal variation in tick host-questing activity, represented by a bimodal curve, shows a significant relationship with microclimatic conditions (particularly temperature) in the *I. ricinus* habitat (Daniel et al., 2015). The first and always considerably higher activity peak corresponds with an average temperature range of 10.1–15.0°C while the second, substantially smaller peak corresponds with an average temperature range of 15.1–20.0°C. However, this pattern changed significantly in 2003 when the numbers of questing nymphs were significantly reduced. Interestingly, a similar anomaly was observed in the 2003 seasonal regression lines comparing the numbers of TBE cases: unlike other years, there were more cases per given number of questing ticks during spring-summer compared with summer–autumn (Figure 4). These coincident changes in the typical seasonal dynamics of questing ticks and TBE incidence followed record-breaking flooding in August 2002 in Central Europe, and coincided with an extremely dry period from March to the end of September in 2003 when temperatures exceeded the 30-year monthly averages by 2.9–4.1°C (Hladný et al., 2004; Menne and Ebi, 2006; Rebetez et al., 2009; Daniel et al., 2015). The correlation between extreme meteorological events and TBE incidence has not previously been reported.

Ambient temperature has a significant influence on the development and activity of arthropod vectors, and on the pathogens they transmit. Most studies of ambient temperature effects on arbovirus infection of vectors have involved mosquitoes rather than ticks (Danielová, 1975; Watts et al., 1987; Mellor and Leake, 2000; Dohm et al., 2002; Carpenter et al., 2011). The effects of temperature (15 and 24°C) and relative humidity (75 and 97% RH) on TBEV-infected *I. ricinus* ticks were examined in laboratory studies using 920 nymphs fed on viraemic laboratory mice (Danielová et al., 1983; Danielová, 1990). The highest infection rates (70 and 73%) were observed at 24°C + 75% RH and 24°C + 97% RH, respectively. Furthermore, nymphs that fed on viremic mice 1–2 months after molting showed 59% infection rates whereas older nymphs (3–4 months after molting from larvae) feeding on the same mice had infections rates of only 29% (*p* < 0.001), suggesting that physiological age affects infection levels. Given that spring host-questing nymphs are those metamorphosed in the preceding year and overwintered as unengorged nymphs while summer-autumn host-questing nymphs are predominantly nymphs metamorphosed in the same summer (Daniel and Dusbábek, 1994), a combination of physiological age and environmental temperature may influence TBEV replication in ticks and account for higher relative numbers of human TBE cases in the summer-autumn season. Studies on the Far Eastern subtype of TBEV in its principal vector, *Ixodes persulcatus*, provide similar indications that extrinsic climatic conditions affect virus infection of the tick vector (Korenberg and Kovalevskii, 1994; Korenberg, 2000). Russian authors also conclude that clinical disease occurs most often when humans are bitten by ticks carrying a relatively high virus dose (Korenberg and Kovalevskii, 1994, 1999; Penyevskaya, 2008). In a study of 1,496 adults and 345 children (all unvaccinated) bitten by adult *I. persulcatus* in a focus of TBEV transmission, the ticks were removed and classified according to the level of infectious virus they carried (Penyevskaya, 2008). Of those bitten by ticks with low virus titers (~1.0 log_10_ TCID_50_), 4/1108 (0.4%) adults, and 2/240 (0.8%) children became symptomatically infected. Ticks with moderate infections (~2.17 log_10_ TCID_50_) were associated with 6/147 (4.1%) adults and 5/44 (11.4%) children symptomatically infected while ticks with high infection levels (~3.08 log_10_ TCID_50_) were associated with 34/241 (14.1%) adults and 20/61 (32.8%) children that developed TBE.

Given that higher temperatures are associated with higher levels of TBEV infection in ticks and with higher incidences of TBE in humans, environmental temperature explains the discrepancy reported here between tick activity and number of cases of TBE in spring-summer compared with summer-autumn periods. Although comparatively frequent in the spring period, the bites by *I. ricinus* ticks (particularly nymphs) during the sharp explosive onset of their host-questing activity (also the start of human outdoor leisure activities) cause relatively fewer cases of clinical disease in comparison with the summer period. Lower average temperatures in the tick habitat may constrain TBEV replication after overwintering such that the threshold dose needed for clinical infection in humans is not attained. In spring, humans bitten by infected ticks may receive only a sub-threshold dose of TBEV causing subclinical (asymptomatic) infection resulting in an immune response. This would explain, for example, the high prevalence of antibodies in individuals in South Bohemia without any previous clinical disease (Luňáčková et al., 2003). Higher average temperatures during the summer-autumn period may lead to higher levels of TBEV in ticks and consequently an increased risk that humans develop the disease following an infected tick bite. Our working hypothesis can be tested by extensive statistical comparison of virus infection levels in ticks collected in the same locality during the spring-summer and summer-autumn periods. Although the logistics of such an undertaking are formidable, given the very low TBEV infection rate in ticks in nature (estimated as <0.1%) modern sequencing technologies now make this challenge tractable (Daniel et al., 2008, 2016).

Daytime ambient temperature directly affects *I. ricinus* host-questing activity, the basis for prognoses of general tick attack risk. This daily warning, based on routine meteorological forecasts, has been implemented in the Czech Republic since 2007 as part of the awareness campaign for inhabitants in TBE risk areas (Daniel et al., 2015). However, conditions affecting TBEV load in tick bites are better represented by weekly average temperatures. Thus the model of general tick attack risk (based on daytime temperature) supplemented with weekly average temperature provides the opportunity for real time forecasts of TBE clinical infection risk as part of an effective campaign to prevent TBE in humans.

## Ethics statement

The study uses national statistics on recorded cases of TBE that do not refer to individual case details and are freely available to academic researchers. Use of these data is exempt from requiring ethical consent.

## Author contributions

MD, VD conceived and designed the study; AF, MM, and BK analyzed the data. PN, MD, and VD wrote the paper.

### Conflict of interest statement

The authors declare that the research was conducted in the absence of any commercial or financial relationships that could be construed as a potential conflict of interest.
