# Supplementary material for: Increased Relative Risk of Tick-Borne Encephalitis in Warmer Weather
Source: Front Cell Infect Microbiol. 2018 Mar 22;8:90. doi: 10.3389/fcimb.2018.00090 (PMC5874492; doi:10.3389/fcimb.2018.00090)
Supplement: Supplementary file 1 [file DataSheet1.DOC]

**Increased risk of tick-borne encephalitis virus in warmer weather**

**Supplementary Material**

**Supplementary Table 1. Comparison of the regression lines representing the relationship between abundance of host-questing *Ixodes ricinus* nymphs and cases of TBE in spring-summer and summer-autumn.**

| **Year** | **Spring-summer** | | **Summer-autumn** | | **Difference of slopes** | **p-value** |
| --- | --- | --- | --- | --- | --- | --- |
|  | **Intercept** | **Slope** | **Intercept** | **Slope** |  |  |
| 2001 | 1.88 | 0.16 | 1.34 | 0.37 | 0.21 | 0.060 |
| 2002 | 0.62 | 0.38 | 0.20 | 0.53 | 0.15 | 0.185 |
| 2003 | 2.02 | 0.40 | 0.10 | 0.53 | 0.13 | 0.311 |
| 2004 | 0.91 | 0.20 | 0.65 | 0.41 | 0.21 | 0.044 |
| 2005 | 0.93 | 0.20 | 0.86 | 0.41 | 0.21 | 0.005 |
| 2006 | 1.11 | 0.31 | 1.02 | 0.65 | 0.34 | 0.005 |
| Total | 0.89 | 0.38 | 0.66 | 0.62 | 0.24 | <0.001 |

**Supplementary Table 2. Comparison of total numbers of questing nymphs and corresponding numbers of calendar weeks in spring-summer and summer-autumn periods at different temperature ranges during 2001-2006.**

Temperatures were derived from near-ground measurements in the tick monitoring site, standard day temperatures, and weekly average temperatures.

| **Temperature measurement** | **Temperature**  **(°C)** | **Spring-summer** | | **Summer-autumn** | |
| --- | --- | --- | --- | --- | --- |
|  |  | **weeks** | **nymphs** | **weeks** | **nymphs** |
| Near-ground |  |  |  |  |  |
|  | ≤0 | 8 | 360 | 0 | 0 |
|  | 0.1-5 | 16 | 1304 | 2 | 119 |
|  | 5.1-10 | 14 | 1351 | 22 | 1463 |
|  | 10.1-15 | 17 | 2128 | 42 | 3349 |
|  | 15.1-20 | 8 | 1147 | 19 | 1341 |
|  | >20 | 3 | 613 | 17 | 1683 |
|  | Total | 66 | 6903 | 102 | 7955 |
| Standard day |  |  |  |  |  |
|  | ≤0 | 1 | 0 | 0 | 0 |
|  | 0.1-5 | 9 | 435 | 0 | 0 |
|  | 5.1-10 | 15 | 1467 | 4 | 202 |
|  | 10.1-15 | 22 | 2723 | 14 | 1132 |
|  | 15.1-20 | 16 | 1883 | 45 | 3003 |
|  | >20 | 3 | 395 | 39 | 3618 |
|  | Total | 66 | 6903 | 102 | 7955 |
| Weekly average |  |  |  |  |  |
|  | ≤0 | 0 | 0 | 0 | 0 |
|  | 0.1-5 | 6 | 201 | 0 | 0 |
|  | 5.1-10 | 14 | 751 | 1 | 52 |
|  | 10.1-15 | 30 | 4124 | 20 | 1285 |
|  | 15.1-20 | 13 | 1599 | 52 | 4190 |
|  | >20 | 3 | 228 | 29 | 2428 |
|  | Total | 66 | 6903 | 102 | 7955 |
